# Undiagnosed cardiovascular risk factors in overweight and obese individuals: a low income country experience

**DOI:** 10.7717/peerj.10870

**Published:** 2021-02-02

**Authors:** Patricio Alfredo Vallejo-Valdivieso, Graciela Zambrano-Pincay, Alberto Ortiz

**Affiliations:** 1 Facultad de Ciencias de la Salud, Universidad Técnica de Manabí, Portoviejo, Ecuador; 2 Nephrology and Hypertension, Instituto de Investigacion Sanitaria Fundacion Jimenez Diaz Universidad Autonoma de Madrid, MADRID, Madrid, España

**Keywords:** Obesity, Overweight, Global burden of disease, Diabetes, Albuminuria, Chronic kidney disease, Low-income, Epidemiology, Undiagnosed, Hypertension

## Abstract

**Background:**

Overweight and obesity are associated with diabetes, hypertension and chronic kidney disease (CKD). However, there is scarce information from lower income countries about undiagnosed obesity-associated conditions. This information is necessary for healthcare planning and for assessment of Global Burden of Disease.

**Methods:**

We assessed the prevalence of obesity-associated conditions in 656 overweight (*n* = 360) and obese (*n* = 296) adults from inner-city Portoviejo (Ecuador), in descriptive field research, based on an opportunistic and selective sampling strategy.

**Results:**

Of 316 men and 340 women, 73% met criteria for prehypertension (27%) or hypertension (46%), 50% met criteria for prediabetes (30%) or diabetes (20%), 11% had an estimated glomerular filtration rate (eGFR) <60 ml/min/1.73 m^2^ consistent with chronic kidney disease (CKD) and 5.5% had pathological albuminuria for a total CKD prevalence of 16%. Age-related prevalence data were generated. In all participants, serum total cholesterol and triglycerides were >200 and >150 mg/dl, respectively. Hyperuricemia and microhematuria (<2%) were uncommon. Women were more likely to have low eGFR (18 vs 5%, *p* 0.000). Diabetes and pathological albuminuria prevalence were higher in obese than in overweight participants (15 vs 12%, *p* 0.018; and 8 vs 4%, *p* 0.0199, respectively).

**Discussion:**

In conclusion, undiagnosed hypertension, diabetes and CKD were more common than expected in overweight and obese persons from Ecuador. Detection rates exceeded official estimates of prevalene of these conditions. Screening the overweight/obese for these conditions, especially at the age ranges at higher risk, may be cost-effective to identify a high number of persons who may benefit from early inexpensive intervention.

## Introduction

Overweight, obesity, diabetes, hypertension and chronic kidney disease (CKD) are non-communicable diseases with increasing prevalence and contribution to the Global Burden of Disease (Foreman et al., 2018). These conditions increase cardiovascular and premature death risk over the long-term while safe, inexpensive interventions improve outcomes (Shlipak et al., 2020). Moreover, these conditions are interrelated. Overweight and obesity favor the development of diabetes, hypertension and CKD, while diabetes and hypertension are the most common causes of CKD (Foreman et al., 2018; Kramer et al., 2020). Thus, the early identification of overweight and obesity related cardiovascular risk factors is key for the early treatment that prevents cardiovascular disease. Indeed, recent conferences have explored how to best achieve early identification of CKD, for example (Shlipak et al., 2020). The epidemiology of obesity and associated conditions is very well characterized in high-income countries, but less information is available from lower income countries. Specifically, there is scarce information on obesity and subclinical associated conditions i.e., those that are undiagnosed and under the radar of healthcare in low income countries. Global efforts, like the Global Burden of Disease study, rely on available information sources to estimate the epidemiology of non-communicable diseases for regions without comprehensive information sources (Foreman et al., 2018; Kramer et al., 2020; GBD 2019 Diseases and Injuries Collaborators, 2020; GBD 2019 Risk Factors Collaborators, 2020; GBD 2019 Demographics Collaborators, 2020). Moreover, local authorities in resource-limited settings require local information on risk factor epidemiology that allows the design and implementation (e.g., selection of target population) of cost-effective screening strategies for the early identification of treatable conditions. In Ecuador, 6 out of 10 adults are overweight or obese (OPS/OMS, 2014). Thus, 43% of men and 38% of women are overweight while 28% of women and 17% of men are obese. Additionally, 80,000 to 120,000 persons are known to have diabetes, and around 50,000 have prediabetes. The Public Health Ministry (MSP) also estimates that 760,000 persons have hypertension and 190,000 pre-hypertension (OPS/OMS, 2014). In 2019, cardiovascular disease contributed 3,040 age-standardized disability-adjusted life-years (DALYs) per 100,000 in Ecuador, while diabetes and CKD contributed 1,200 and 1,100, respectively (GBD 2019 Diseases and Injuries Collaborators, 2020). These three conditions contributed 21% of total DALYs. Additionally, high body-mass index (BMI), high fasting plasma glucose, high blood pressure and kidney dysfunction, in this order, were the leading risk factors for DALYs and for deaths in 2019 in Ecuador and their negative impact continued to increase from 2010 to 2019 (GBD 2019 Risk Factors Collaborators, 2020). Early identification and adequate management of these risk factors may contribute to decrease burden of disease and healthcare costs and increase the life-expectancy at birth, currently estimated at 76.4 years (GBD 2019 Demographics Collaborators, 2020). We have now explored the epidemiology of undiagnosed conditions related to overweight/obesity in a random sample from an inner-city quarter of the city of Portoviejo in Ecuador. The results suggest that the prevalence of diabetes and hypertension may be higher than previously estimated and that screening for diabetes, hypertension and CKD in the overweight/obese population may lead to a high yield in the early identification of these conditions. Moreover, the results characterized the age ranges in which screening may increase the yield of positive findings.

## Materials & Methods

This was an exploratory, descriptive field research, based on an opportunistic and selective strategy in a population sample from Parroquia Andres de Vera, in the city of Portoviejo. Portoviejo is the 8th most populous city in Ecuador and the Portoviejo canton had 280.029 inhabitants in 2010 (Resultados del censo, 2020). Parroquia Andres de Vera in an inner city, low-income quarter, one of the 13 parishes and one of the seven urban parishes of the Portoviejo canton. Portoviejo is the capital of the province of Manabí, located in the central zone of the Ecuadorian coast, in the northwest of the country.

Information was collected between November 1, 2018 and September 1, 2019. Random homes in this parish were visited and 1,389 people were interviewed, of which the 656 who met the following inclusion criteria were selected: Sign the informed consent, age 18 or older and overweight or obese, according to the WHO definition (BMI ≥25.0 kg/m2). According to data from the National Institute of Statistics and INEC Census, in 2016, the population of Parroquia Andres de Vera was 90,225 people, 43,895 men and 46,330 women (Mieles Giler, 2020), of which approximately 60% were over the age of 18 years (Anonymous, 2020). Thus, for this study, approximately 2.6% of the Parroquia Andres de Vera was approached, and 1.2% agreed to participate and met study entry criteria. After obtaining the informed consent, additional information on overweight/obesity and health implications was provided and instructions were provided for further assessments.

### Variables

The patients who met the inclusion criteria underwent a physical examination (height, weight, blood pressure), and blood and urine analyses.

The physical examination included blood pressure measured after 30 min of rest by means of an aneroid sphygmomanometer Rudolf Riester GmbH Mod. Big Ben® on three different days. The most repeated, and, in its absence, alternatively the highest value was recorded, as this screening exercise aimed at being sensitive. From height and weight, the body mass index was calculated and overweight and/or obese patients were categorized. Participants produced a 24 h urine sample and fasting capillary glycemia was determined using the Accu-Check® Active III (Roche Diagnostics) and blood was drawn for serum creatinine, urea, uric, total cholesterol and triglycerides in serum. Serum and urine parameters were assessed twice with one-month interval and the mean of the two values was calculated. Age was collected as categorical values in 5-year intervals and the mean of the age interval was used to estimate glomerular filtration rate (eGFR) from serum creatinine using the CKD-EPI equation (Levey et al., 2009). Albuminuria was assessed in 24 h urine samples by the microalbuminuria latex assay kit (Biosystems, Barcelona, Spain). Urine density was assessed by a densitometer and pH using reactive strips (pH-FIX 0-14), also in 24 h urine samples. Hematuria was assessed by microscopy or urinary sediment.

The following definitions were used: overweight (BMI 25.0–29.9 kg/m2) and obesity (≥30.0 kg/m2) were defined as per the WHO. Fasting glycemia between 100 and 125 mg/dl was considered prediabetes and ≥126 mg/dl diabetes, as per the American Diabetes Association  (American Diabetes Association, 2020). Normal blood pressure was considered as systolic blood pressure (SBP) <120 and diastolic blood pressure (DBP) <80 mm Hg, pre-hypertension (SBP 120/129 and DBP <80 mm Hg), grade I hypertension (SAD 130/139 and SAD 80/89 mm Hg) and hypertension grade II (TAS>140 and TAD>90 mm Hg) following the American Heart Association (AHA) in 2017 (Whelton et al., 2018).

Albuminuria was categorized according to the KDIGO Guidelines into A1 (<30 mg/24 h), A2 (30–300 mg/24 h) and A3 (>300 mg/24 h) and CKD was defined, also following KDIGO, as eGFR <60 ml/min/1.73 m2 or A2/A3 albuminuria (Perez-Gomez et al., 2019). However, the 3-month persistence of pathological findings was not required, as is the case for most cross-sectional studies of CKD prevalence (Brück et al., 2016).

Hypertriglyceridemia was considered when values were >150 mg/dl and hypercholesterolemia when total serum cholesterol were above 200 mg/dl and hyperuricemia when serum urate was >7.0 mg/dl in men and >6.0 mg/dl in women while microhematuria was defined as >3 red cells per field, all according to the normal range in the clinical laboratory.

Persons with normal glycemia and eGFR; 3 records of normal blood pressure; and absence of pathological albuminuria in the 24-hour urine tests, did not have further follow-up or counselling, while medical monitoring was recommended for patients with prediabetic/diabetic capillary blood glucose testing, blood pressure compatible with prehypertension and / or hypertension, or pathological albuminuria or eGFR.

### Statistical analysis

The statistical analysis was performed with the SPSS Program (Statistical Package for the Social Sciences), version 24. Quantitative data are reported as mean ± standard deviation or median (interquartile range) as appropriate, and categorical data as *n* (%). Student’s *t* test was used to assess differences between normally distributed quantitative variables and Mann–Whitney *U* test for non-normally distributed variables from two groups and Chi square to assess differences in categorical variables. A two-sided *p* < 0.05 was considered to be statistically significant.

### Ethics and institutional review board statement

The study was conducted according to the guidelines of the Declaration of Helsinki, and approved by the Research Ethics Committee of the Autonomous University of Madrid (protocol code CEI-101-1899, October 14, 2019), with the signing of the informed consent of the patients who participated as a population sample and the authorization of the MSP through the Health Center Andres de Vera (protocol code MSP-CZ4-13D01-DDS-2018-0992-O, August 30, 2018). Informed consent was obtained from all subjects involved in the study.

## Results

Out of 656 participants, 360 (54.9%) were overweight and 296 (45.12%) obese. Among overweight patients, 184 (51.11%) were men and 176 (48.88%) women. Among obese patients, 132 (44.59%) were men and 164 (55.41%) women. Overall mean age was 53.0  ± 22.4 years and was not different between the 316 men and 340 women (Table 1).

**Table 1:** Clinical data according to gender. Data expressed as *n* (%), mean ± standard deviation or median (interquartile range). A two-sided *p* < 0.05 was considered to be statistically significant and is shown in bold. Within non- statistically significant (ns) cells, only numerical values less than 0.1 are shown. Student’s *t* test was used to assess differences between normally distributed quantitative variables and Mann-Whitney *U* test for non-normally distributed variables from two groups and Chi square to assess differences in categorical variables within each row.

	**All**, *n* = 656	**Men**, *n* = 316	**Women**, *n* = 340	***p* value**
Age (years)	53.01 ± 22.4	54.63 ± 21.70	51.51 ± 22.91	ns (0.074)
BMI (kg/m^2^)	30.88 ± 4.29	30.70 ± 4.32	31.04 ± 4.28	ns
Overweight, *n* (%)	360 (54.88%)	184 (58.22%)	176 (51.76%)	ns (0.096)
Obese, *n* (%)	296 (45.12%)	132 (41.77%)	164 (48.23%)	
SBP (mmHg)	128.84 ± 11.41	129.04 ± 11.50	128.65 ± 11.34	ns
DBP (mmHg)	82.81 ± 6.89	82.92 ± 6.98	82.70 ± 6.81	ns
Prehypertension/hypertension	476 (72.56%)	230 (72.78%)	246 (72.35%)	ns
Prehypertension	176 (26.83%)	81 (25.65%)	95 (27.94%)	ns
Hypertension	300 (45.73%)	149 (47.15%)	151 (44.41%)	ns
Glycemia (mg/dl)	102.80 ± 19.60	104.48 ± 19.70	101.24 ± 19.41	**0.034**
Prediabetes/diabetes	325 (49.54%)	166 (52.53%)	159 (46.76%)	ns
Prediabetes	196 (29.88%)	93 (29.43%)	103 (30.29%)	ns
Diabetes	129 (19.66%)	73 (23.10%)	56 (16.47%)	**0.033**
Albuminuria, mg/24 h	27.60 (26.75–28.35)	27.53 (26.80–28.30)	27.65 (26.65–28.40)	ns
Albuminuria ≥30 mg/24 h, *n* (%)	36 (5.49%)	14 (4.43%)	22 (6.47%)	ns
Urea (mg/dL)	32.60 ± 3.3	32.47 ± 3.27	32.71 ± 3.37	ns
Creatinine (mg/dL)	0.91 ± 0.17	0.97 ± 0.17	0.85 ± 0.15	**<0.0001**
eGFR (ml/min/1.73 m^2^)	85.12 ± 20.7	88.37 ± 18.98	82.11 ± 21.71	**<0.0001**
eGFR <60 ml/min/1.73 m^2^	78 (11.28%)	16 (5.06%)	62 (18.2%)	**<0.0001**
eGFR <60 ml/min/1.73 m^2^ or albuminuria ≥30 mg/24 h	106 (16.15%)	30 (9.49%)	76 (22.35%)	**<0.0001**
Urate (mg/dl)	4.57 ± 0.96	5.16 ± 0.85	4.03 ± 0.69	**<0.0001**
Hyperuricemia^a^	6 (0.19%)	3 (0.94%)	3 (0.88%)	ns
Cholesterol total (mg/dL)	220 ± 12	220 ± 12	220 ± 12	ns
Triglycerides (mg/dL)	173 ± 14	173 ± 14	172 ± 13	ns
Urine density	1.029 ± 0.004	1.029 ± 0.003	1.029 ± 0.004	ns
Urine pH	6.0 (5.0–7.0)	6.0 (5.0–7.0)	6.0 (6.0–7.0)	ns
Urine RBC (*n* per field)	1.0 (1.0–2.0)	1.0 (0.0–2.0)	2.0 (1.0–3.0)	ns
Microhematuria^b^	13 (1.98%)	5 (1.58%)	8 (2.35%)	ns

**Notes.**

SBP systolic blood pressure DBP systolic blood pressure eGFR estimated glomerular filtration rate

Systolic blood pressure. Fasting glycemia between 100 and 125 mg/dl was considered prediabetes and ≥126 mg/dl diabetes.

a >6.0 mg/dL in women, >7.0 mg/dL in men.

b >3 RBC per field.

**Figure 1:**
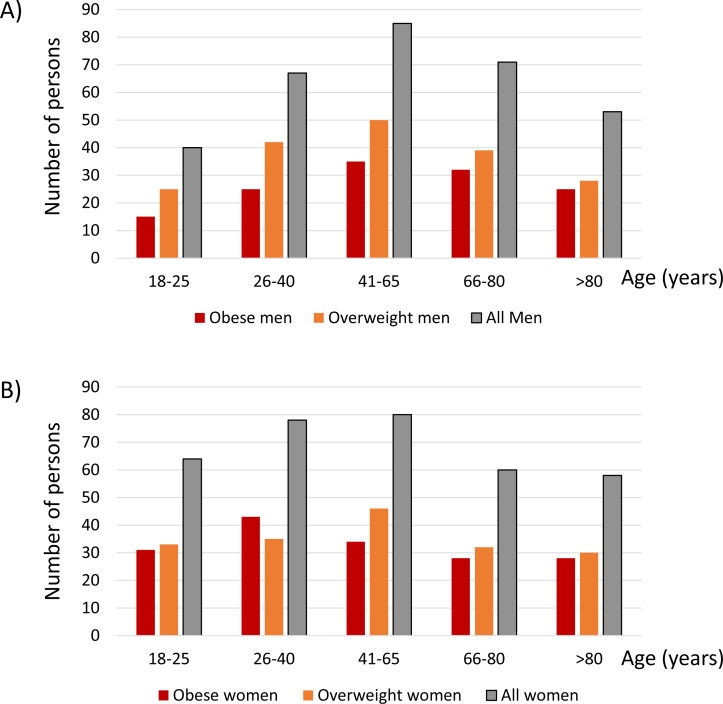
Age distribution for men (A) and women (B). The number of participants for each age category is shown.

The age distribution for men (Fig. 1A) and women (Fig. 1B) is shown in Fig. 1 for all, obese and overweight participants. The most represented age group was 41–65 years of age in both men and women: 27% of men and 24% of women were in this age group.

Table 1 also shows other study variables for all and for men and women separately. Overall SBP was 128.84 ± 11.41 mmHg and DBP 82.81 ± 6.89 mmHg, both above normal limits, and 73% of participants had either pre-hypertension (27%) or hypertension (46%). Mean glycemia was 102.80 ± 19.60 mg/dl and 50% of participants had either prediabetes (30%) or diabetes (20%). Median albuminuria values were 27.60 (26.75–28.35) mg/24 h and 5.5% of patients had pathological albuminuria (i.e., albuminuria ≥ 30 mg/24 h). All of these participants had category A2 albuminuria (30–300 mg/24 h) and there were no participants with higher (A3) albuminuria. Albuminuria >100 mg/24 h was present in 22 (3.3%) participants. Mean eGFR was 85 ± 21 ml/min/1.73 m2 and 11% of participants had eGFR <60 ml/min/1.73 m2, consistent with KDIGO CKD category G3, as no participant had eGFR <30 ml/min/1.73 m2. Overall, 16% of participants met either eGFR or albuminuria numerical criteria for eGFR. In all participants, serum total cholesterol and triglycerides were >200 and >150 mg/dl, respectively. Hyperuricemia and microhematuria (<2%) were uncommon.

Significant differences between men and women included higher serum urate, higher glycemia (104 ± 20 vs 101 ± 19 mg/dl; *p* = 0.034) and prevalence of diabetes (23 vs 16%, *p* 0.0328) and eGFR (88 ± 19 vs 82 ± 22 ml/min/1.73 m2, *p* 0.000), in men, while in men there was a lower prevalence of CKD (9 vs 22%, for CKD defined as low eGFR or high albuminuria, *p* = 0.000) (Table 1).

Table 2 presents data for obese and overweight participants overall and for men and women separately.

The main differences between obese and overweight participants, in addition to a higher BMI in obese participants (35 ± 3 vs 27.5 ± 1.5 kg/m2, *p* 0.000) were a higher prevalence of diabetes (25 vs 12%, *p* = 0.018) and of pathological albuminuria (8 vs 4%, *p* = 0.0199) in obese than in overweight participants: The highest diabetes prevalence was observed in obese men (31%) and the lowest in overweight women (13%) while the highest CKD prevalence was observed in obese females (23%) and the lowest in overweight men (7%). Differences in hypertension prevalence for different subgroups were not as striking.

**Figure 2:**
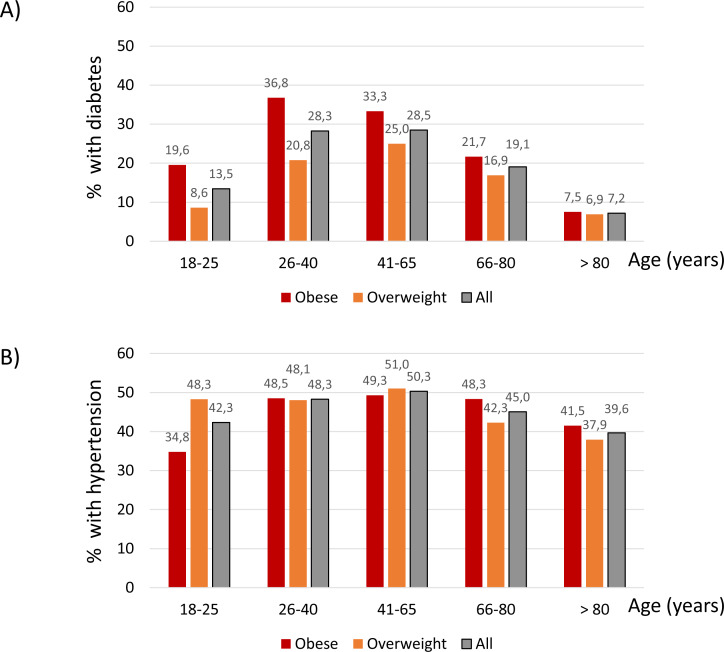
Percentage of participants with diabetes (A) and hypertension (B) within each age category.

Regarding age categories, the highest prevalence of diabetes was observed in 26–40-year-old obese participants (37%), closely followed by 41–65-year-old obese participants (33%) (Fig. 2A). These age ranges also corresponded to the highest prevalence of diabetes in overweight participants (21 and 25%, respectively) (Fig. 2A).

Hypertension followed a similar age-related pattern, although differences between age groups were not as marked as for diabetes. The peak prevalence of hypertension was observed in 41-65-year-old obese (49%) and overweight participants (51%) (Fig. 2B).

**Table 2:** Clinical data according to presence of overweight or obesity. Data expressed as *n* (%), meanstandard deviation or median (interquartile range). A two-sided *p* < 0.05 was considered to be statistically significant and is shown in bold. Within non-statistically significant (ns) cells, only numerical values less than 0.1 are shown. Student’s *t* test was used to assess differences between normally distributed quantitative variables and Mann-Whitney *U* test for non-normally distributed variables from two groups and Chi square to assess differences in categorical variables within each row.

	**Overweight**				**Obese**				***P* value obese vs overweight (all)**
	**All**, *n* = 360	**Men**, *n* = 184	**Women**, *n* = 176	***p***^c^	**All**, *n* = 296	**Men**, *n* = 132	**Women**, *n* = 164	*p*^c^	
Age (years)	52.78 ± 22.19	53.26 ± 21.66	52.28 ± 22.78	ns	53.29 ± 22.63	56.54 ± 21.69	50.68 ± 23.10	**0.027**	ns
BMI (kg/m^2^)	27.51 ± 1.46	27.50 ± 1.44	27.51 ± 1.48	ns	34.98 ± 2.75	35.16 ± 2.73	34.83 ± 2.78	ns	**<0.0001**
SBP (mmHg)	128.78 ± 11.20	129.77 ± 11.54	127.74 ± 10.77	ns (0.086)	128.91 ± 11.68	128.03 ± 11.41	129.62 ± 11.87	ns	ns
DBP (mmHg)	82.69 ± 6.94	81.89 ± 6.67	83.47 ± 7.13	**0.031**	82.95 ± 6.83	82.17 ± 6.71	83.57 ± 6.88	ns (0.078)	ns
Prehypertension/hypertension	261 (72.5%)	137 (74.46%)	124 (70.45%)	ns	215 (72.63%)	93 (70.45%)	122 (74.39%)	ns	ns
Prehypertension	95 (28.39%)	42 (22.82%)	53 (30.11%)	ns	81 (27.37%)	39 (29.55%)	42 (25.61%)	ns	ns
Hypertension	166 (46.11%)	95 (51.63%)	71 (40.34%)	**0.032**	134 (45.27%)	54 (40.91%)	80 (48.78%)	ns	ns
Glycemia (mg/dl)	101.73 ± 18.94	103.37 ± 18.94	100.00 ± 18.83	ns (0.092)	104.10 ± 20.34	106.03 ± 20.69	102.56 ± 19.99	ns	ns
Prediabetes/diabetes	173 (48.05%)	96 (52.17%)	77 (43.75%)	ns	152 (51.35%)	70 (53.03%)	82 (50.0%)	ns	ns
Prediabetes	118 (32.77%)	64 (34.78%)	54 (30.68%)	ns	78 (26.4%)	29 (21.97%)	49 (29.88%)	ns	ns (0.074)
Diabetes	55 (12.28%)	32 (17.4%)	23(13.06%)	ns	74 (25.0%)	41 (31.1%)	33 (20.12%)	**0.031**	**0.002**
Albuminuria, mg/24 h	29.94 ± 14.76	29.54 ± 13.35	30.35 ± 16.13	ns	36.85 ± 37.2	35.71 ± 34.59	37.76 ± 39.27	ns	**0.001**
Albuminuria ≥30 mg/24 h, *n* (%)	13 (3.61%)	6 (3.26%)	7 (3.98%)	ns	23 (7.77%)	8 (6.06%)	15 (9.15%)	ns	**0.020**
Urea (mg/dL)	32.70 ± 3.02	32.68 ± 2.98	32.72 ± 3.08	ns	32.47 ± 3.66	32.18 ± 3.64	32.70 ± 3.66	ns	ns
Creatinine (mg/dL)	0.92 ± 0.17	0.98 ± 0.17	0.85 ± 0.15	**<0.0001**	0.90 ± 0.16	0.96 ± 0.16	0.86 ± 0.15	**<0.0001**	ns
eGFR (ml/min/1.73 m^2^)	85.34 ± 20.3	88.28 ± 18.56	82.27 ± 21.66	**0.005**	84.85 ± 21.1	88.49 ± 19.62	81.93 ± 21.82	**0.008**	ns
eGFR <60 ml/min/1.73 m^2^	41 (11.39%)	8 (4.35%)	33 (18.75%)	**<0.0001**	37 (12.50%)	8 (6.06%)	29 (17.68%)	**0.003**	ns
eGFR <60 ml/min/1.73 m^2^ or albuminuria ≥30 mg/24h	51 (14.17%)	13 (7.06%)	38 (21.59%)	**<0.0001**	55 (18.58%)	17 (12.88%)	38 (23.17%)	**0.024**	ns
Urate (mg/dl)	4.57 ± 0.97	5.13 ± 0.85	3.99 ± 0.71	**<0.0001**	4.58 ± 0.94	5.21 ± 0.85	4.07 ± 0.66	**<0.0001**	ns
Hyperuricemia^a^	2 (0.56%)	1 (0.54%)	1 (0.57%)	ns	4 (1.35%)	2 (1.52%)	2 (1.22%)	ns	ns
Cholesterol total (mg/dL)	220 ± 12	220 ± 12	219 ± 12	ns	220 ± 12	220 ± 12	220 ± 12	ns	ns
Triglycerides (mg/dL)	172 ± 14	173 ± 14	171 ± 13	ns	173 ± 14	173 ± 13	173 ± 14	ns	ns
Urine density	1.029 ± 0.003	1.029 ± 0.003	1.028 ± 0.003	**0.042**	1.029 ± 0.004	1.029 ± 0.004)	1.029 ± 0.004	ns	ns
Urine pH	6.0 (5.0–7.0)	6.0 (5.0–7.0)	6.0 (5.0–7.0)	ns	6.0 (5.0–7.0)	6.0 (5.0–7.0)	6.0 (6.0–7.0)	ns	ns
Urine RBC (*n* per field)	2.0 (1.0–2.0)	1.0 (0.0–2.0)	2.0 (1.0–3.0)	**0.006**	1.0 (1.0–3.0)	1.50 (1.0–3.0)	1.0 (0.0–2.0)	ns	ns
Microhematuria^b^	11 (3.06%)	4 (2.18%)	7 (3.98%)	ns	2 (0.68%)	1 (0.76%)	1 (0.61%)	ns	**0.045**

**Notes.**

SBP systolic blood pressure DBP systolic blood pressure eGFR estimated glomerular filtration rate

Systolic blood pressure. Fasting glycemia between 100 and 125 mg/dl was considered prediabetes and ≥126 mg/dl diabetes.

a >6.0 mg/dL in women, >7.0 mg/dL in men.

b >3 RBC per field.

c Men vs women within subgroup.

**Figure 3:**
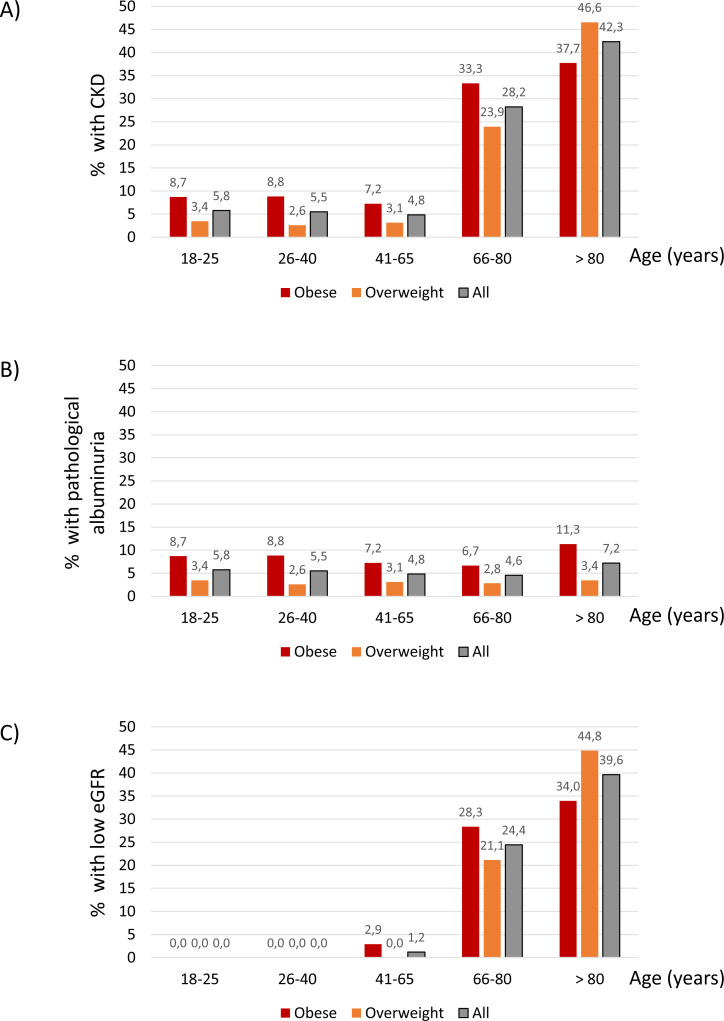
CKD. Percentage of participants with CKD (eGFR < 60 ml/min/1.73 m^2^ or albuminuria ≥30 mg/24 h); (A) pathological albuminuria ( ≥30 mg/24 h) (B) or low eGFR (eGFR < 60 ml/min/1.73 m^2^) (C) within each age category.

The age distribution of CKD differed from that of diabetes and hypertension. Thus, CKD prevalence increased sharply from 4.85% at age 41–65 years to 28.24 at age 66–80 years and 42.34% from age 80 years and higher (Fig. 3A). This was the result of different age-related patterns for the two components of the CKD definition. Thus, the prevalence of pathological albuminuria was higher for obese participants at all ranges than for overweight participants (Fig. 3B) and there was no observable increase in prevalence with age. By contrast low GFR was not observed below the age 41 and was very uncommon in the 41-65-year age range (Fig. 3C). This is consistent with findings in the general population worldwide. Additionally, obesity or overweight-related hyperfiltration may have obscured any loss of kidney mass at younger age ranges.

## Discussion

The main findings of the present study are that undiagnosed prehypertension, hypertension, prediabetes, diabetes and pathological albuminuria were highly prevalent in overweight and obese persons from Ecuador, likely more common than previously suspected, given the official figures for obesity, diabetes and hypertension. Additionally, a high prevalence of CKD diagnosed as low eGFR was observed in the older patients and as pathological albuminuria at all ages in the obese. These findings provide the groundwork for future screening strategies followed by dietary and health advice as well as eventual inexpensive pharmacological interventions that may reverse the growing impact on DALYs of the four key risk factors in Ecuador, namely, increased BMI, hyperglycemia, high blood pressure and kidney dysfunction (GBD 2019 Risk Factors Collaborators, 2020).

The 2020 Ecuador population is estimated at around 10 million adults  (Central Intelligence Agency, 2020), of which 60% are overweight or obese (OPS/OMS, 2014), resulting in around 6 million overweight or obese persons. If we hypothesize that our data can be extrapolated to the whole Ecuadoran population, this would result in over 1 million diabetics and over 2.5 million hypertensive persons. These numbers are 10-fold and 3-fold higher, respectively, than official estimates (OPS/OMS, 2014), but are aligned with global diabetes prevalence estimates (Saeedi et al., 2019). Thus, the present study identified a high-risk population and age-ranges in which screening for diabetes, hypertension and associated complications, such as CKD, may be cost effective. Cost-effectiveness may be increased by decreasing the number of tests performed. Thus, assessment of urea, uric acid and urinalysis other than albuminuria did not significantly increase the detection of treatable conditions.

The peak prevalence of diabetes and hypertension was observed in middle age, rather than in the older participants. Being a cross-sectional study, we can only speculate to the drivers of this observation, which differs from the increasing hypertension and diabetes prevalence with age in most high-income countries (Saeedi et al., 2019; Burt et al., 1995). We interpret this as representing a negative impact of these conditions (diabetes, hypertension, CKD) on the life expectancy of younger obese and overweight participants.

The present study served to recommend participants with abnormal findings to seek medical advice. This may be based on simple lifestyle changes (exercise, diet, stop smoking) or be further supported, once the diagnoses were confirmed, by drug therapy. Renin-angiotensin system blockade and metformin represent low cost and effective drugs for hypertension, kidney protection, diabetes treatment and weight loss  (Fernandez-Fernandez et al., 2020). While SGLT2 inhibitors are yet expensive, the cost is expected to significantly come down in the near future, providing additional drugs that treat type 2 diabetes while promoting weight loss, a lowering blood pressure and affording kidney and cardiac protection (Fernandez-Fernandez et al., 2019). An additional intervention may consist of increasing water intake; Thus, the urine density was relatively high for a 24 h urine collection, potentially denoting insufficient water intake to maintain ADH values low. In this regard, the local climate may favor continuous water losses through skin. High ADH levels have been associated with CKD progression, most notably in so-called Mesoamerican nephropathy and in polycystic kidney disease, that is now treated with an ADH receptor V2 blocker, tolvaptan (Carriazo et al., 2019; Perez-Gomez et al., 2018).

Some limitations should be acknowledged. Thus, there is no non-obese, non-overweight control group. This is because the focus of research were cardiovascular risk factors in overweight-obese individuals. However, because of this design, this study does not provide information on the prevalence of overweight and obesity. Additionally, only total cholesterol values are available and no information on LDL- or HDL-cholesterol values was obtained. Although for some patients eGFR was below 60 ml/min/1.73 m^2^, as eGFR was assessed from the mean of two serum creatinine values separated one month, the 3-month persistence criterion to diagnose CKD was not met. However, as already indicated, this is also the case for most cross-sectional studies on CKD epidemiology. In this regard, as a strength compared to other epidemiological study, serum parameters and albuminuria were assessed twice with one-month interval, thus decreasing potential interference with any subclinical acute condition. Finally, another limitation, consequence of field work in a low-resource scenario, was the lack of glycated hemoglobin data. Finally, Manabi province may have specific characteristics not extrapolatable to the rest of Ecuador. According to the Encuesta Nacional de Salud y Nutrición (ENSANUT) 2018, Manabí province has a prevalence of overweight of 40% among adults aged 19 to 59 years, which is close to the national average of 41% and an obesity ‘prevalence of 29%, higher than the national average of 23%, for a combined overweight/obesity prevalence of 69% (Mendoza et al., 2021). This represents an increase from 38% and 24% (combined 61%) prevalence of overweight/obesity in 2012, at a time when the national average was 41 and 22% (combined 63%) respectively (Freire et al., 2021). These data are complemented by the Encuesta sobre Salud, Bienestar y Envejecimiento (SABE) in adults 60-year-old or older (Orces & Gavilanez, 2017). which found an age-adjusted prevalence of abdominal obesity in Ecuador of 61% to 87%, of hyperglycemia of 39% and 51% and of hypertension of 58 to 61%, while the absolute prevalence of diabetes was 13% and 20% in men and women respectively. Manabí cuisine is rich in carbohydrates from green banana, peanut and corn and local water quality maybe suboptimal as drinking water was reported to contain traces of pesticides and being very hard (Bravo Moreira, 2013).

## Conclusions

In conclusion, undiagnosed hypertension, diabetes, and CKD were common in overweight and obese persons from Ecuador and detected rates far exceed official estimates. In this regard, extrapolation of the present data to the whole Ecuadoran population would result in over 1 million diabetics and over 2.5 million hypertensive persons, 10-fold and 3-fold higher, respectively, than official estimates, but in line with global diabetes prevalence estimates. The fact that the findings fall within global estimates of prevalence support the validity of the findings and suggest that they should be the basis for the design of population wide, cost-effective screening strategies. The present study additionally identified the ranges at which the prevalence of these conditions is higher in this high-risk population, thus facilitating the design of such screening studies in search of diabetes, hypertension and associated complications, such as CKD, that are cost effective and allow early, unexpensive pharmacological and non-pharmacological interventions.

##  Supplemental Information

10.7717/peerj.10870/supp-1 Data S1 Raw data

## Additional Information and Declarations

### Data Availability

The following information was supplied regarding data availability: Data are available in the Supplemental Files.
